# Expanding the knowledge about Thauvin-Robinet-Faivre syndrome: a case report with novel clinical findings and review of the literature

**DOI:** 10.1007/s13353-025-00984-2

**Published:** 2025-06-19

**Authors:** Andrea Cosentino, Flavia D’Orazio, Roberto Magnato, Wilhelm Berger

**Affiliations:** 1 Department of Orthopedics and Traumatology, Franz Tappeiner Hospital, via Rossini 5, Meran, 39012 Bolzano, Italy; 2 Paracelsus Medical University, Strubergasse 21, 5020 Salzburg, Austria; 3 Department of ENT – Maxillofacial Surgery, Franz Tappeiner Hospital, via Rossini 5, Meran, 39012 Bolzano, Italy

**Keywords:** TROFAS, FIBP gene, Overgrowth, Developmental delays, Hearing loss

## Abstract

This case report expands the phenotypic spectrum of Thauvin-Robinet-Faivre syndrome (TROFAS, OMIM #617107), a rare autosomal recessive disorder caused by biallelic loss-of-function mutations in the *FIBP* gene. We describe a patient with genetically confirmed TROFAS who presented with novel clinical features, including non-ossifying fibromas, subglottic tracheal stenosis, intermediate uveitis, and complete atrioventricular block requiring pacemaker implantation. The findings significantly broaden the phenotypic landscape of TROFAS and underscore the need for multidisciplinary management and long-term follow-up.

## Introduction

Thauvin-Robinet-Faivre syndrome (TROFAS, OMIM #617107) is an ultra-rare autosomal recessive condition caused by mutations in the *FIBP* gene on chromosome 11q13.1. The *FIBP* gene encodes the FGF1 intracellular binding protein, which modulates fibroblast growth factor (FGF) signaling, a key pathway in embryonic development and tissue homeostasis.

TROFAS is characterized by tall stature, intellectual disability, and renal abnormalities, among other congenital anomalies. Overgrowth syndromes comprise a group of genetic disorders defined by excessive growth, manifesting as increased height and weight (Weaver 1994; Neylon et al. 2012). These conditions typically result in growth and physical development parameters exceeding two standard deviations above the mean for the individual’s age and sex. The mechanisms responsible for overgrowth phenotypes have been linked to an increased cell count resulting from heightened cellular proliferation, hypertrophy, or a combination of both. To investigate the underlying cause of overgrowth and assess the pathogenicity of the identified mutation, a previous study (Chung et al. 2012) analyzed the proliferation rate of cultured fibroblasts. Notably, fibroblasts derived from patient skin samples exhibited a significantly higher proliferation rate compared to controls. Similar findings were reported by Thauvin-Robinet et al. (2016), who suggested that the detected indel mutation exerts a loss-of-function effect on the FIBP protein. These results further reinforce the role of FIBP as a key regulator of growth.

Clinically, TROFAS is characterized by generalized overgrowth, particularly in height, macrocephaly, intellectual disability ranging from mild learning difficulties to severe developmental delays, facial dysmorphism, and various congenital anomalies, including cardiac and renal malformations. Notably, there is an increased risk for Wilms tumor in affected individuals.

**Fig. 1:**
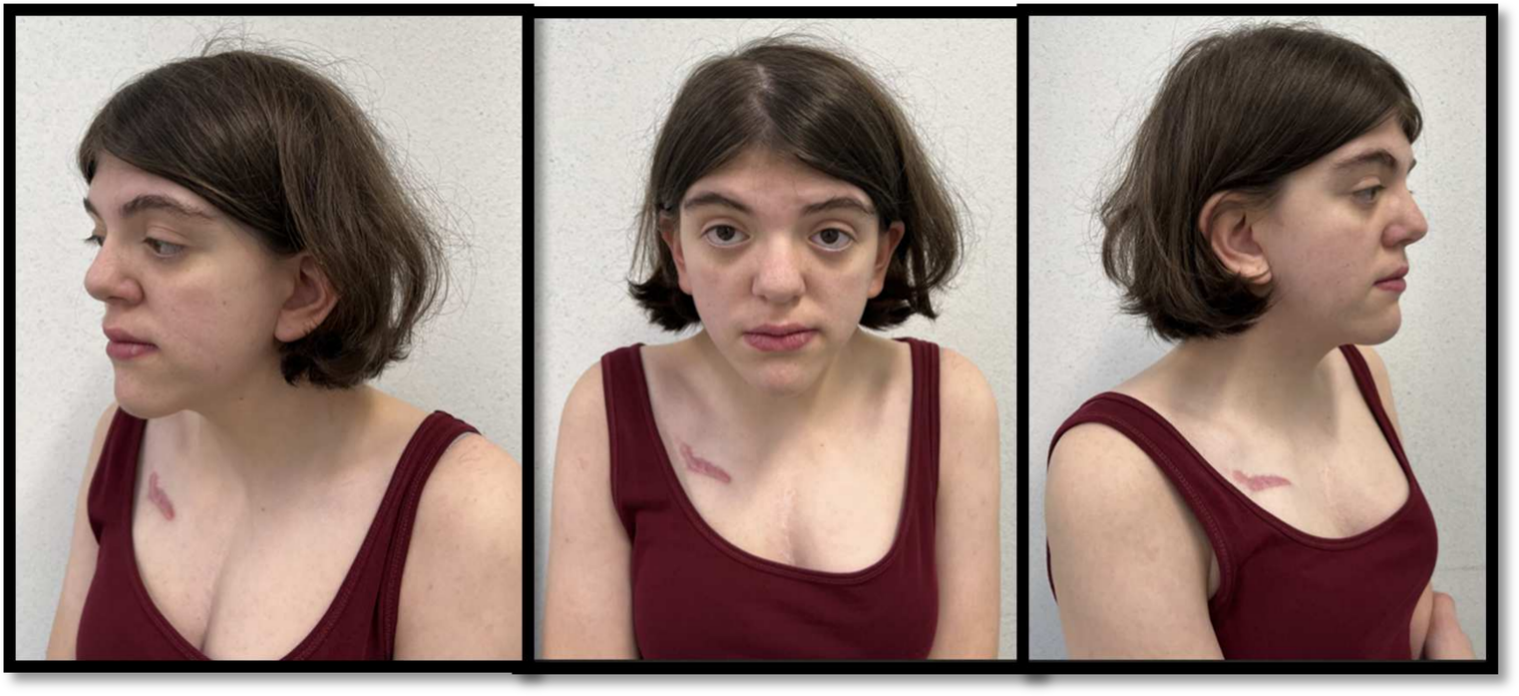
Fenotipical aspect of the patient

Given the rarity of TROFAS, with only a handful of cases reported in the literature, each new case provides valuable insights into the phenotypic spectrum and potential complications associated with the syndrome. Here, we present a genetically confirmed case of TROFAS, highlighting novel clinical features not previously reported in the literature. Notably, references to functional assays on fibroblasts previously reported by other authors have been included for context and were not performed by the present team.

## Materials and methods

### Case presentation

The patient is a 14-year-old Caucasian female with confirmed TROFAS. She measured 161 cm and weighed 60 kg (both corresponding to approximately P55/+1.2 standard deviation score (SDS) and P70/+1.5 SDS for her age, respectively) and a BMI of 23.15. The patient is the second child of non-consanguineous parents, and the brother appears healthy. During the pregnancy, reduced fetal movements were noted and a spontaneous vaginal delivery occurred at 36th+4 weeks of gestation. At birth, the infant had a weight of 2420 gs (25th–50th percentile), a length of 49 cm (50th–75th percentile), and a head circumference of 32 cm (10th–25th percentile). Apgar scores were 8 at 1 min, 9 at 5 min, and 10 at 10 min (Fig. 1).

Developmental milestones were delayed, with independent walking achieved at approximately 2 years of age and delayed speech development noted. She began school at the age of 7 and required the support of a caregiver during the first year of middle school. According to the end-of-year school report, her cognitive abilities and academic skills were not age-appropriate. She required consistent routines in daily school activities and had difficulty adapting to changes or new demands. Notable challenges included reduced memory, increased distractibility, and impaired concentration. Additionally, she struggled with handling money, navigating traffic, and appropriately assessing dangerous situations.

At the age of six, the patient underwent to some genetic tests. Chromosomal analysis revealed a normal female karyotype: 46,XX (based on the evaluation of 50 metaphases). Interphase fluorescent in situ hybridization on buccal mucosa cells was performed to investigate a possible Turner mosaicism, with unremarkable findings. FISH analysis was also performed from the peripheral blood to rule out a microdeletion in the chromosomal region 22q11.2, using the DiGeorge/VCFS TUPLE1 Region Probe (Cytocell), with the karyotype reported as ish 22q11.2(HIRAx2). However, in all examined metaphases, both chromosome 22 showed specific hybridization signals for the HIRA (TUPLE1) gene as well as the control signals. Therefore, a microdeletion in the investigated chromosomal region could be ruled out with high probability. After several years without a definitive diagnosis, exome analysis at the age of 13 identified two heterozygous mutations in the *FIBP* gene: c.1003C>T, p.(Arg335*), and c.497_498del, p.(Ser166*). Parental testing revealed that each parent carries one of these mutations: the mother is heterozygous for the c.497_498del mutation, while the father is heterozygous for the c.1003C>T mutation. This confirms that the patient has inherited one mutated allele from each parent, resulting in a compound heterozygous state for the *FIBP* gene.

**Fig. 2:**
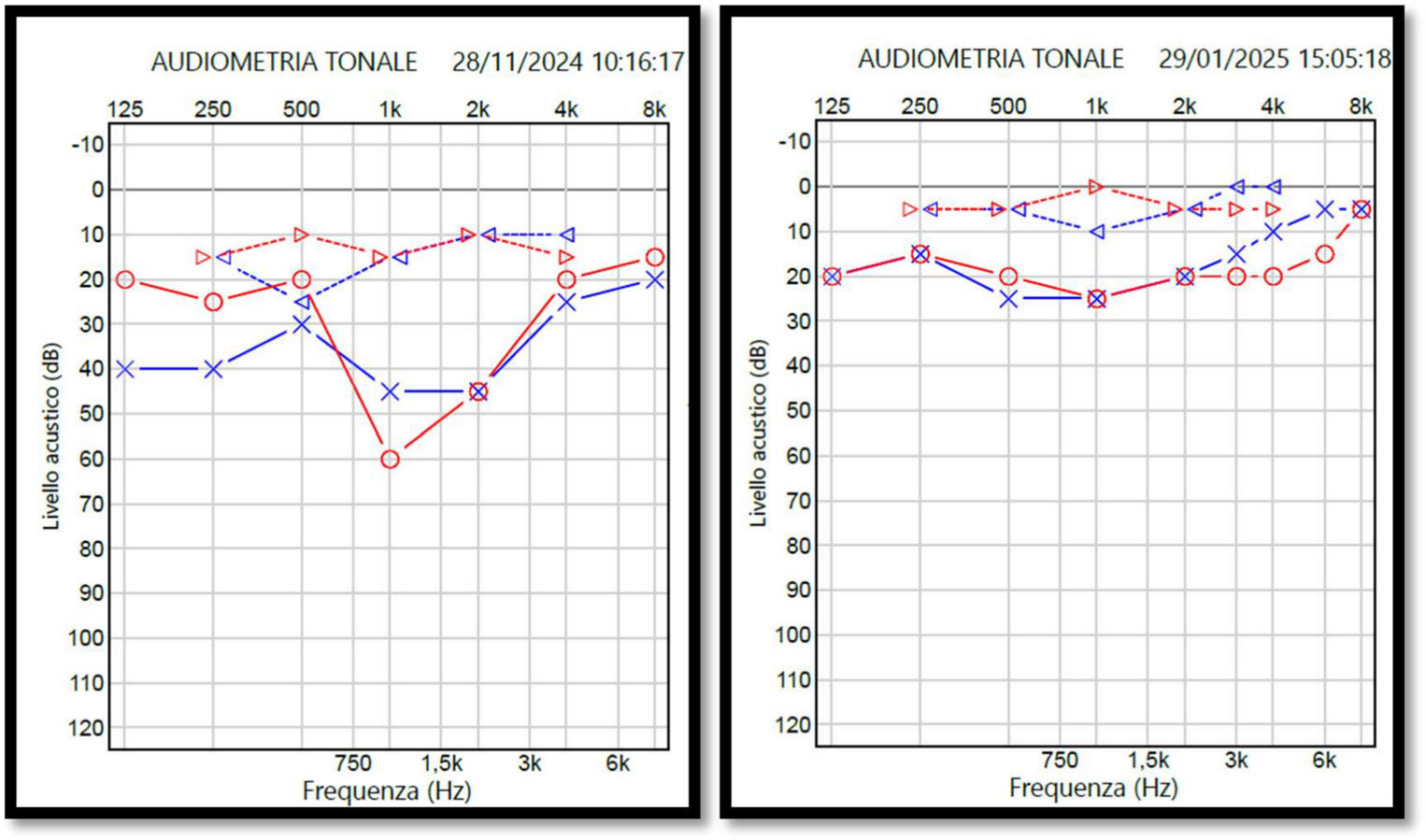
Tonal audiometry showing a fluctuating transmission hearing loss due to ear ventilatory dysfunction

Pulmonary complications have been a significant issue, as the patient experienced recurrent respiratory infections, including bronchitis and pneumonia, attributed to a hypoplastic pulmonary artery. In July 2024, a bronchoscopy revealed a 50% obstruction of the main bronchus, leaving the left lung nonfunctional. Lung fibrosis was also noted, which further complicated her respiratory status.

The patient’s otolaryngological history is notable for subglottic tracheal stenosis, which has significantly contributed to her respiratory challenges. Since early childhood, she has experienced intermittent conductive hearing loss due to tubal dysfunction, without any evidence of sensorineural impairment. Figure 2 shows two tonal audiometry performed 2 months apart in which the right (red lines) and left (blue lines) transmission hearing loss changes at the level of aerial auditory perception (bottom line), which is due to tubal and thus ventilatory dysfunction.

Endoscopically, she presents bilateral hypertrophy of the Torus tubarius and grade III tonsillar hypertrophy (tonsils are beyond the pillars), despite undergoing an adeno-tonsillotomy in 2015 to alleviate obstructive symptoms and recurrent infections. Her tympanic membranes remain intact and free of sclerosis. Notably, she does not exhibit signs of sleep apnea. Dental evaluation has revealed a posterior crossbite malocclusion.

Her cardiac history is notable for congenital heart anomalies, including a double-chambered right ventricle, a patent foramen ovale, and a ventricular septal defect, all surgically repaired in January 2011. Additionally, the patient has left pulmonary vein atresia and a progressively enlarging mediastinal vascular conglomerate, predominantly peritracheal and left peribronchial, which has been documented since 2018. In June 2024, she developed a complete atrioventricular block, necessitating the implantation of a pacemaker in July 2024.

**Fig. 3:**
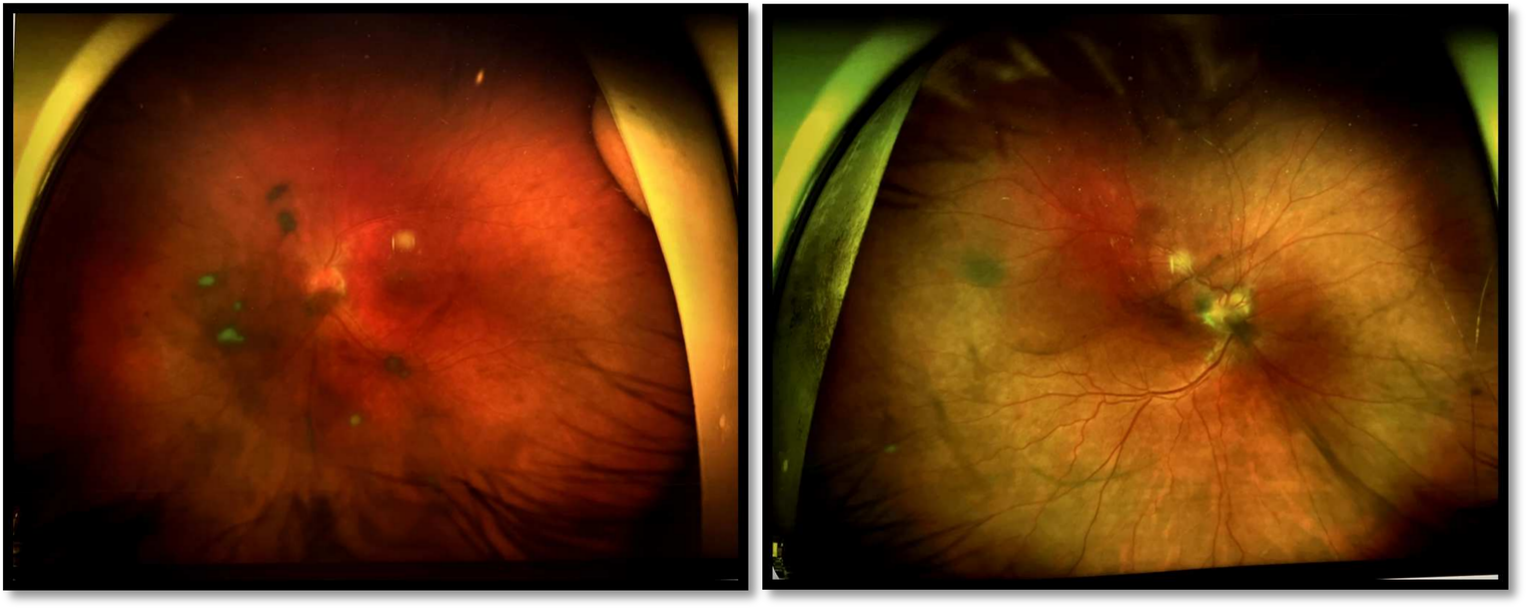
Left: vitritis with snowballs in the left eye. Right: arterial anomaly

The patient also presents significant ophthalmologic findings. She was diagnosed with intermediate uveitis, for which she began Adalimumab therapy in December 2023. By October 2023, she developed increasing vitritis with snowballs in the left eye, while a known arterial anomaly in the right eye has remained stable (Fig. 3). Other causes of uveitis and AV block were excluded.

Additionally, a significant vitamin D deficiency was identified, warranting rheumatologic evaluation and supplementation. At birth, the patient presented with a left clubfoot deformity, which was managed conservatively with the redressive casts and a tenotomy of the Achilles tendon, according with Ponseti method (Heck et al. 2016).

**Fig. 4:**
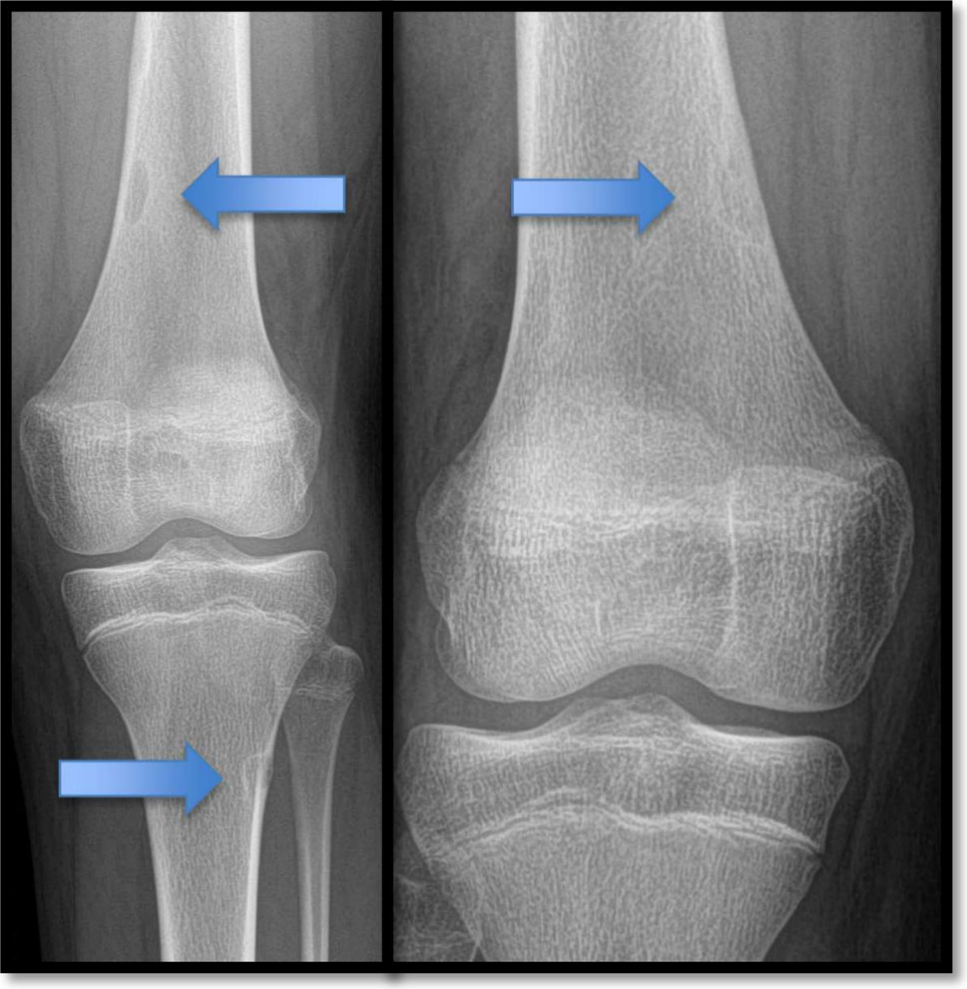
RX with arrows show the non-ossifying fibromas of the left and right knee

**Fig. 5:**
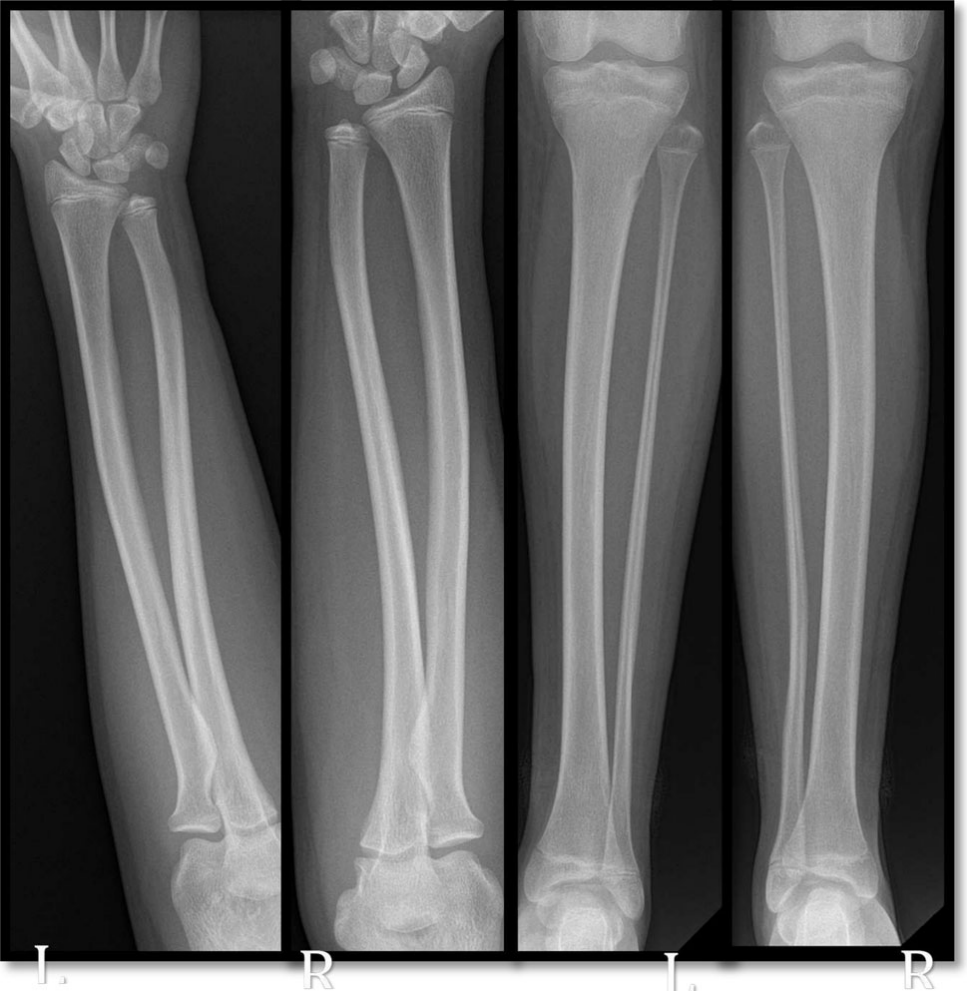
RX of arm and leg show the bowing of radius, ulna, tibia, and fibula

In November 2024, she reported bilateral knee pain, and radiographic evaluation revealed benign, non-ossifying fibromas in both knees (Fig. 4), a finding not previously associated with TROFAS. Radiographical findings of the upper and lower extremities show the bowing of both sides of the tibia, radius and ulna, probably associated with low levels of vitamin D (Fig. 5).

## Discussion

TROFAS is an exceedingly rare genetic disorder, with limited cases documented in medical literature. The syndrome’s phenotypic spectrum is broad, encompassing various congenital anomalies and developmental delays. This case contributes to the expanding understanding of TROFAS by highlighting several novel clinical manifestations (Table 1).

The present study broadens the clinical spectrum of TROFAS by documenting previously unreported manifestations, particularly in the musculoskeletal, respiratory, cardiac, and neuropsychiatric domains. The discovery of non-ossifying fibromas (NOFs) in the knees, presenting as bilateral knee pain, represents a novel feature in TROFAS patients. While NOFs are common benign bone lesions in children and adolescents, their presence in this context suggests a potential genetic predisposition linked to abnormal bone remodeling processes. Skeletal anomalies such as scoliosis, tibial bowing, and metatarsal varus deformities have been reported in TRFS cases, but the occurrence of NOFs indicates an additional aspect of aberrant bone homeostasis that has not been previously described (Kılıç and Koşukcu 2024). Given that FIBP mutations disrupt fibroblast growth factor (FGF) signaling, a pathway critical for bone differentiation and growth, it is plausible that these mutations contribute to structural skeletal defects, warranting further investigation (Akawi et al. 2016).

Respiratory challenges in TROFAS extend beyond recurrent infections to include structural anomalies such as subglottic tracheal stenosis and unilateral lung dysfunction due to a hypoplastic pulmonary artery, reinforcing the need for continuous pulmonary monitoring. Prior studies have described chronic pulmonary disease and recurrent respiratory infections as common complications in TROFAS (Duzenli et al. 2023). Given the role of FGF signaling in lung development, it is conceivable that FIBP loss-of-function mutations contribute to congenital airway and vascular malformations, increasing susceptibility to pulmonary compromise (Kılıç and Koşukcu 2024). The presence of hypoplastic pulmonary vasculature further underscores the vasculopathic phenotype of TROFAS, which requires detailed cardiopulmonary surveillance.

**Table 1:** Comparison of clinical features in the present and previously reported TROFAS cases

Clinical features	Present study	Thauvin-Robinet et al. (2016)	Akawi et al. (2016)	Kılıç et al. (2024)
Macrocephaly	+	+	–	+
Tall stature	+	+	+	+
Developmental delay	+	+	+	+
Intellectual disability	+, mild	+	+	+, mild
Hearing loss	+	–	${\pm }$	–
Facial dysmorphic features	+	+	+	+
Round face	–	–	+	${\mp }$
Flat midface	+	–	+	${\mp }$
Widely-spaced eyes	+	–	+	+
Down-slanting palpebral fissures	+	+	–	+
Epicanthic folds	+	–	+	${\mp }$
Thick lips	+	+	+	–
Skeletal abnormalities	Clubfeet, non-ossifying fibromas, bowing of tibia, radius and ulna	Camptodactyly	Talipes-metatarsus varus, bowing of tibia, spina bifida	Scoliosis
Striae on the skin	–	–	–	${\pm }$
Cardiac abnormalities	AV block, double-chambered right ventricle, patent foramen ovale, ventricular septal defect, left pulmonary vein atresia	Ventricular septal defect, mitral valve prolapse	Ventricular septal defect, double-chambered right ventricle	Minimal pericardial effusion
Ocular abnormalities	Intermediate uveitis, vitritis with snowballs	Retinal coloboma	Astigmatism, cataract, strabismus	–
Renal abnormalities	–	Renal malrotation, bifid ureter	Nephromegaly, cystic dysplastic kidney	Atrophic dysplastic kidney
Malignancies	–	–	Wilms tumor	–
Hematologic abnormalities	–	Benign neutropenia	Benign neutropenia	–
*FIBP* gene variant	c.1003C>T, c.497_498del	c.652C>T	c.175_176insTTA	c.412-3_415dupCAGTTTG
Amino acid change	p.(Arg335*), p.(Ser166*)	p.Gln218*	p.His59delinsLeuAsn	p.?

Cardiac complications in TROFAS have been well-documented, but the progression to complete atrioventricular (AV) block requiring pacemaker implantation is a particularly critical finding. While ventricular septal defects, double-chambered right ventricles, and patent foramen ovale have been reported in TROFAS, the development of significant cardiac conduction abnormalities requiring intervention has not been widely described (Kılıç and Koşukcu 2024). Given the widespread expression of FIBP in cardiac tissues, it is plausible that its disruption affects both myocardial development and electrical conduction, which could explain the propensity for arrhythmical complications. This underscores the importance of serial electrocardiographic and echocardiographic evaluations in TROFAS patients.

Neuropsychiatric involvement is an emerging concern in TROFAS. The first documented case of early psychosis in a TRFS patient suggests that FIBP mutations may influence neurodevelopmental pathways, potentially leading to cognitive and psychiatric manifestations (Andreou et al. 2021). A 24-year-old TROFAS patient developed acute psychotic symptoms, including hallucinations, delusions, and behavioral disturbances, necessitating hospitalization and antipsychotic treatment. This raises the possibility of FIBP-related neuropsychiatric vulnerability, possibly mediated through disrupted FGF signaling in cortical and limbic structures. The association between psychosis and overgrowth syndromes has been explored in other genetic disorders, and the identification of psychiatric complications in TROFAS warrants closer monitoring of behavioral and cognitive outcomes in affected individuals.

Ocular involvement in TROFAS continues to expand, with the observation of intermediate uveitis and vitritis with vascular anomalies broadening the known ophthalmologic phenotype. Previous reports have described refractive errors, retinal coloboma, and cataracts, but the presence of uveitis, an inflammatory eye disorder, introduces a novel immunological dimension to TRFS pathophysiology (Duzenli et al. 2023). This finding suggests a possible autoimmune component in TROFAS, warranting further ophthalmologic screening and immunological evaluation in affected patients.

At the molecular level, the FIBP gene, located on chromosome 11q13.1, encodes a fibroblast growth factor (FGF) intracellular binding protein, which modulates cell proliferation, differentiation, and survival. Loss-of-function mutations in FIBP lead to dysregulated FGF signaling, contributing to widespread developmental abnormalities (Akawi et al. 2016). Studies have suggested that FIBP interacts with key transcription factors such as STAT3, regulating gene expression in pathways associated with oncogenesis and cellular overgrowth (Duzenli et al. 2023). This mechanism provides insight into the predisposition to Wilms tumor in TROFAS patients and raises concerns regarding potential malignancy risks, further emphasizing the necessity of oncologic surveillance.

## Conclusion

In conclusion, this study enriches the clinical understanding of Thauvin-Robinet-Faivre Syndrome by documenting previously unreported orthopedic, cardiac, pulmonary, and ophthalmologic manifestations. The identification of non-ossifying fibromas, AV block, and intermediate uveitis underscores the expanding phenotypic landscape of this rare disorder. Given the involvement of FIBP in fundamental growth and signaling pathways, further research is essential to elucidate the molecular mechanisms driving these clinical features. Establishing comprehensive surveillance protocols, including musculoskeletal evaluations, cardiology assessments, pulmonary function monitoring, and ophthalmologic screenings, will be critical in optimizing care and improving outcomes for individuals affected by TROFAS.

## Data Availability

Upon request of the editor.

## Declarations

### Disclaimer

The authors confirm that they have followed ethical guidelines, including protecting patient privacy; for the better understanding of the clinical findings, we did not anonymize the face of the girl. During the preparation of this work, the authors used ChatGPT to improve readability and language. After using this tool, the authors reviewed and edited the content as needed and take full responsibility for the content of the publication.
